# The temporal effects of topical NF-*κB* inhibition, in the *in vivo* prevention of bile-related oncogenic mRNA and miRNA phenotypes in murine hypopharyngeal mucosa: a preclinical model

**DOI:** 10.18632/oncotarget.27706

**Published:** 2020-09-01

**Authors:** Dimitra P. Vageli, David Kasle, Sotirios G. Doukas, Panagiotis G. Doukas, Clarence T. Sasaki

**Affiliations:** ^1^The Yale Larynx Laboratory, Department of Surgery, Yale School of Medicine, New Haven, CT, USA

**Keywords:** NF-κB inhibition, BAY 11-7082, bile, laryngopharyngeal reflux, hypopharyngeal cancer

## Abstract

Supraesophageal bile reflux at strongly acidic pH can cause hypopharyngeal squamous cell cancer, through activation of the oncogenic NF-κB-related pathway. We hypothesize that topical pre- or post-application of pharmacologic NF-κB inhibitor, BAY 11-7082 (0.25 μmol), on murine (C57BL/6J) HM (twice a day for 10 days) can effectively inhibit acidic bile (10 mmol/l; pH 3.0) induced oncogenic molecular events, similar to prior *in vitro* findings. We demonstrate that the administration of BAY 11-7082, either before or after acidic bile, eliminates NF-κB activation, prevents overexpression of *Bcl2, Rela, Stat3, Egfr, Tnf, Wnt5a,* and deregulations of *miR-192*, *miR-504*, linked to bile reflux-related hypopharyngeal cancer. Pre- but not post-application of NF-κB inhibitor, significantly blocks overexpression of *Il6* and prostaglandin H synthases 2 (*Ptgs2*), and reverses *miR-21, miR-155, miR-99a* phenotypes, supporting its early bile-induced pro-inflammatory effect. We thus provide novel evidence that topical administration of a pharmacological NF-κB inhibitor, either before or after acidic bile exposure can successfully prevent its oncogenic mRNA and miRNA phenotypes in HM, supporting the observation that co-administration of NF-κB inhibitor may not be essential in preventing early bile-related oncogenic events and encouraging a capacity for further translational exploration.

## INTRODUCTION

Tobacco, alcohol, and human papilloma virus have been shown to be associated with head and neck cancers [1]. Recent epidemiologic descriptions have also supported the early oncogenic effects of biliary gastroesophageal reflux on the supra-esophageal mucosa, and with an increasing interest in laryngopharyngeal carcinogenesis [2]. Our prior *in vitro* and *in vivo* explorations show the tumorigenic effect of bile at strongly acidic pH on hypopharyngeal cells, enabled through the transcriptional factor NF-*κ*B [3–7].

It has been shown that head and neck cancer exhibits abundant NF-*κ*B activation, and that NF-*κ*B-related oncogenic pathway plays a key role in its pathogenesis [8–13]. During this process NF-*κ*B interacts with a complex network of other cancer related transcriptional factors, cytokines and growth factors and miRNA molecules, including EGFR/Ras/RAF/MAPK, Akt/PI3K/mTOR, ΙΚΚ/NF-κB, STAT3, and wnt/β-catenin, *miR-21, miR-34a, miR-451a and miR-99a* [13–19]. It has also been demonstrated that transcriptional activation of *EGFR, STAT3, BCL2, WNT5A, TNF-α*, and *IL-6*, as well as deregulation of “oncomirs” *miR-21, miR-155, miR-192* and tumor suppressor *miR-375, miR-451a*, *miR-34a, miR-504 and miR-99a* are associated with biliary reflux-related hypopharyngeal squamous cell carcinoma [20], while *in vitro* and *in vivo* explorations have documented that acidic bile-induced oncogenic mRNA and miRNA phenotypes can be prevented by simultaneous co-administration of NF-κB inhibitor in human hypopharyngeal cells [21–24].

We recently showed, using an *in vitro* model, that a pharmacologic NF-*κ*B inhibitor, BAY 11-7082, applied to hypopharyngeal primary cells, before or after exposure to acidic bile, exerted effects comparable to its simultaneous co-application with acidic bile in successfully inhibiting its cancer-related mRNA and miRNA phenotypes [25]. To expand our exploration, we hypothesize that *in vivo* pre- or post- topical exposure of murine hypopharyngeal mucosa to BAY 11-7082, will have similar blocking effects as seen *in vitro*. Determining the *in vivo* temporal characteristics of NF-*κ*B inhibition, using BAY 11-7082, will support future preclinical and clinical trials using NF-*κ*B inhibitors.

## RESULTS

### Inhibition of acidic bile-induced NF-κB activation by *in vivo* topical exposure of HM to BAY 11-7082 either before or after acidic bile application

**Figure 1:**
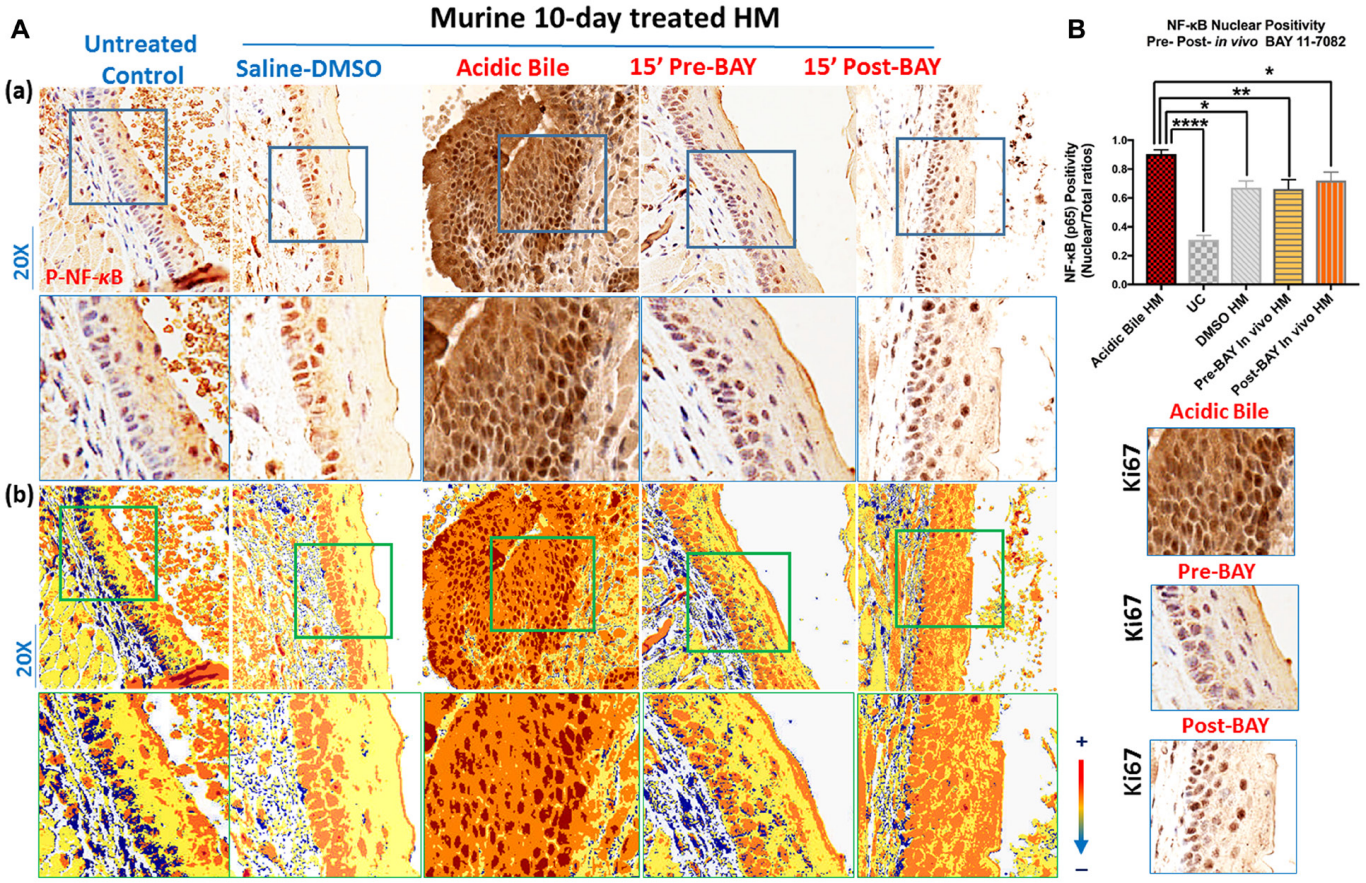
*In vivo* topical pre- or post-application of BAY 11-7082 inhibits the acidic bile-induced NF-*κ*B activation in 10-days exposed murine HM. (**A**) (a) IHC analysis for p-NF-*κ*B (p65 S536) (from left to right): control untreated HM, revealing cytoplasmic staining; saline-DMSO treated HM, demonstrating sporadic and weak cytoplasmic or nuclear staining in a few basal cells; acidic bile-treated HM, producing intense nuclear and cytoplasmic staining of basal and parabasal/suprabasal layers; pre-application inhibitor (BAY 11 7082) + acidic bile-treated HM, demonstrating nuclear or cytoplasmic staining mainly of cells of basal layer and weak cytoplasmic staining of suprabasal layers; acidic bile + post-application inhibitor (BAY 11 7082)-treated HM, demonstrating nuclear or cytoplasmic staining mainly of cells of basal layer and a weak nuclear or cytoplasmic staining of few cells in suprabasal layers; (b) Image analysis algorithm (s) (red: positive nuclear staining of p-NF-*κ*B; orange: intense positive cytoplasmic staining of p-NF-*κ*B; yellow: weak cytoplasmic staining of p-NF-*κ*B; blue: negative p-NF-*κ*B staining) [Images were captured using Aperio CS2 and analyzed by Image Scope software (Leica Microsystems, Buffalo Grove, IL) that generated algorithm (s) illustrating mucosal and cellular compartments demonstrating p-NF-*κ*B staining]. (**B**) Nuclear positivity of p-NF-*κ*B (p65 S536) in murine HM (*P* values by *t* test; multiple comparisons by Holm-Sidak; GraphPad Prism 6.0) (positivity = nuclear-positive/total positive p-p65 staining). Acidic bile (pH 3.0) induces significantly higher positive nuclear p-NF-*κ*B (p65 S536) levels compared to saline-DMSO treated HM or untreated control. Topical pre- or post-application of BAY 11-7082 significantly decreases nuclear p-NF-*κ*B levels in acidic bile (pH 3.0) HM (*p* values by *t* test; multiple comparisons by Holm-Sidak; GraphPad Prism 6.0) (positivity = nuclear-positive/total positive p-p65 staining).

Immunohistochemical analysis revealed that, similar to our prior observations, short-term (10-day) topical application of acidic bile on HM enhanced the activation of NF-*κ*B (Figure 1A and 1B) [24] by intense nuclear staining of p-NF-*κ*B (p65 S536) through mucosal layers (Figure 1A). However, HM treated with NF-*κ*B inhibitor BAY 11-7082 before or after its exposure to acidic bile inhibited NF-*κ*B activation, identified by a weak p-NF-κB (p65 SS536) nuclear staining in mucosal layers compared to HM exposed to acidic bile alone (Figure 1A). Pre-treatment with BAY 11-7082 was found to induce a more intense inhibitory effect of NF-*κ*B activation relative to post-treatment. HM treated by saline-DMSO (control) produced low but not negligible levels of NF-*κ*B activation (Figure 1A), while untreated control showed negative nuclear p-NF-*κ*B staining (Figure 1A).

Scoring of nuclear p-NF-*κ*B (p65 S536) revealed significantly higher levels of activated NF-*κ*B in 10-day exposed HM to acidic bile compared to controls (Figure 1B) (*p* < 0.05, *t* test; mean ± SD; multiple comparisons by Holm-Sidak), similar to our prior findings [24]. In contrast, the effect of pre or post application of BAY 11-7082 significantly reduced NF-*κ*B activated levels relative to acidic bile alone (Figure 1B).

### Inhibition of acidic bile-related Ki67 *in vivo* by topical exposure of HM to BAY 11-7082 either before or after acidic bile

**Figure 2:**
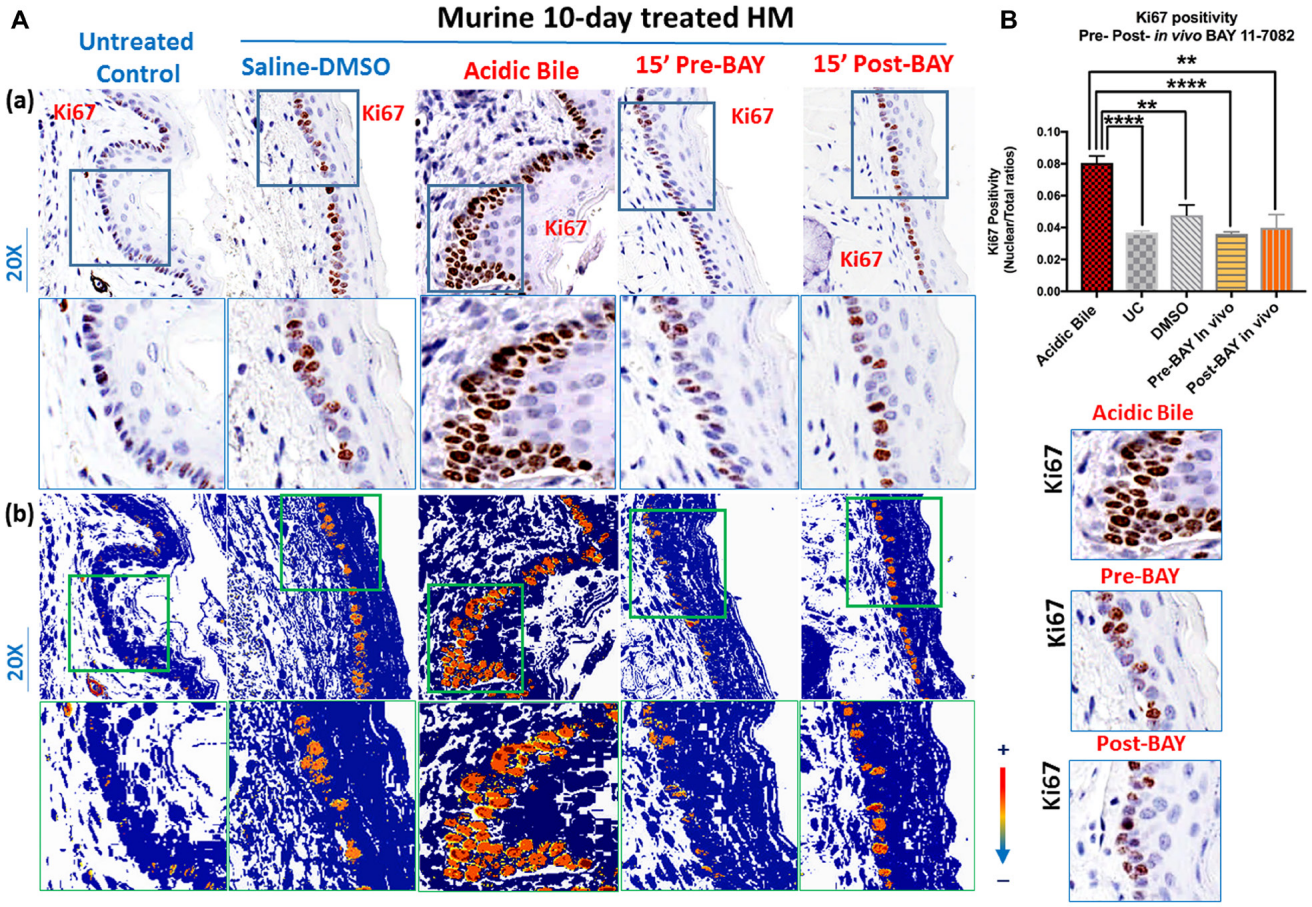
*In vivo* topical pre- or post-application of BAY 11-7082 prevents acidic bile-induced cell proliferation markerKi67 in 10-day exposed murine HM. (**A**) (a) IHC analysis for Ki67 (from left to right): *control untreated HM*, revealing weak cytoplasmic or nuclear staining in few basal cells; *saline-DMSO treated HM*, demonstrating sporadic and weak cytoplasmic or nuclear staining in cells of the basal layer; *acidic bile-treated HM*, revealing intense nuclear and cytoplasmic staining of cells of basal and parabasal layers; *pre-application inhibitor (BAY 11 7082) + acidic bile-treated HM* and *acidic bile + post-application inhibitor (BAY 11 7082)-treated* HM, producing weak nuclear or cytoplasmic staining in cells of the basal layer. (b) Image analysis algorithm (s) (red: nuclear positive staining of Ki67; orange: intense positive cytoplasmic staining of Ki67; yellow: weak cytoplasmic staining of Ki67; blue: negative Ki67 staining) [Images were captured using Aperio CS2 and analyzed by Image Scope software (Leica Microsystems, Buffalo Grove, IL) that generated algorithm (s) illustrating the mucosal and cellular Ki67 staining compartments]. (**B**) Graphs depict 10-day short-term exposure of HM to acidic bile (pH 3.0) inducing significantly higher positive nuclear Ki67 levels compared to saline-treated HM or untreated control. Topical pre- or post-application of BAY 11-7082 significantly decreases nuclear Ki67 levels in acidic bile (pH 3.0) HM (*p* values by *t* test; multiple comparisons by Holm-Sidak; GraphPad Prism 6.0) (positivity = nuclear-positive/total-positive Ki67 staining).

Ten-day exposure of HM to acidic bile induced increased proliferation marker Ki67 in regenerating cells compared to controls (Figure 2A and 2B), similar to our previous findings [24] This was identified by intense Ki67 staining in basal and parabasal layers of acidic bile-treated HM (Figure 2A). However, treatment with BAY 11-7082 before or after acidic bile exposure successfully prevented the acidic bile-induced regeneration of basal cells documented by comparably less intense Ki67 staining (Figure 2A), similar to those levels observed in Saline-DMSO treated HM or untreated controls.

Scoring of nuclear Ki67 showed that application of BAY 11-7082 on HM before or after acidic bile exposure induced significantly lower levels of nuclear Ki67 relative to acidic bile alone (Figure 2B) (*p* < 0.05, *t* test; mean ± SD; multiple comparisons by Holm-Sidak). Conversely, acidic bile alone induced higher levels of nuclear Ki67 levels relative to controls (saline-DMSO treated or untreated HM), similar to our prior findings [24].

We note that microscopic examination of hematoxylin and eosin (H&E) stained treated-HM did not reveal histological signs of toxicity after 10-day local treatment.

### Inhibition of acidic bile-induced upregulation of the NF-κB-related oncogenic mRNA profile by *in vivo* topical exposure of HM to BAY 11-7082 either before or after acidic bile exposure

**Figure 3:**
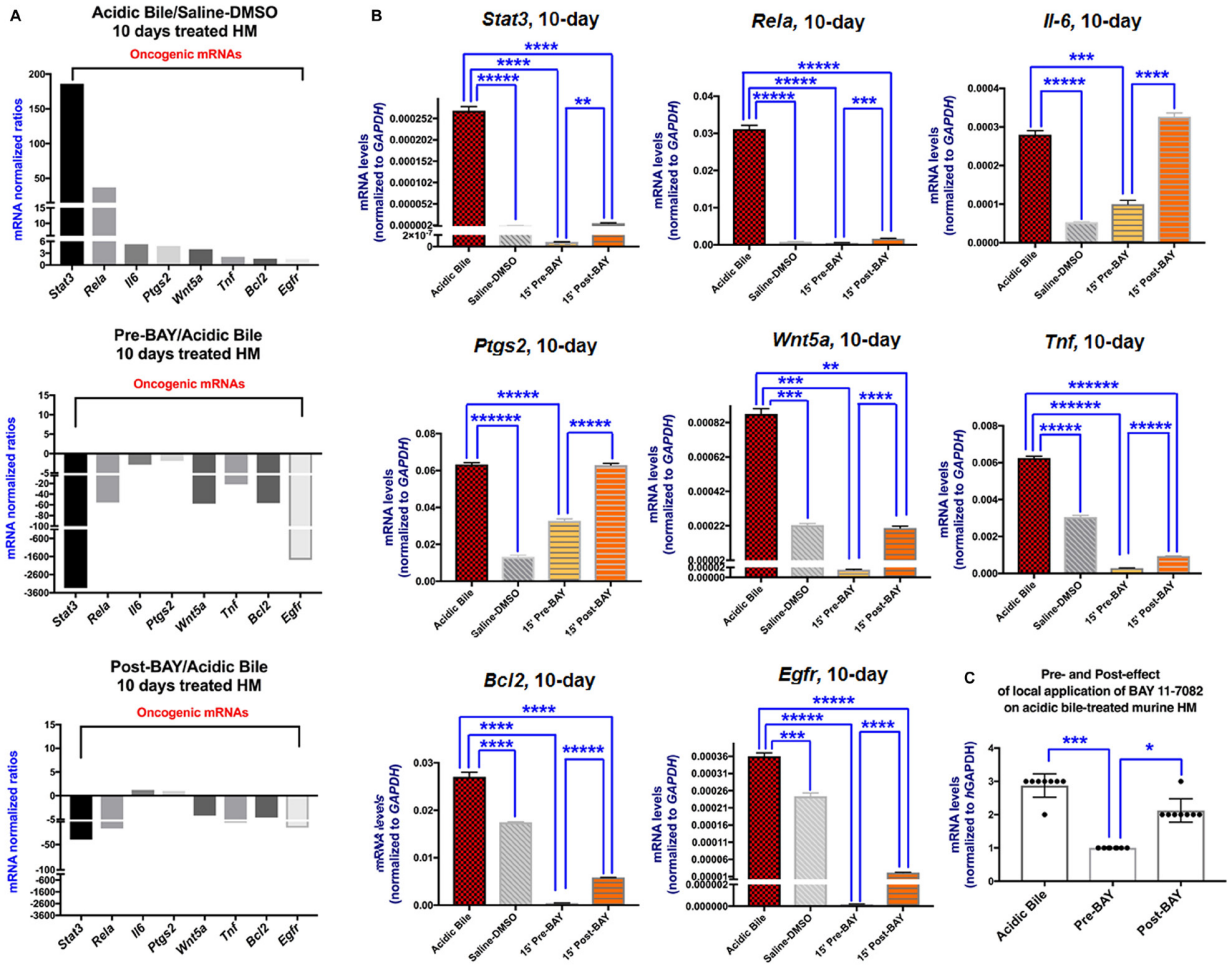
*In vivo* topical pre- or post-application of BAY 11-7082 prevents the acidic bile-induced transcriptional activation of NF-*κ*B related genes with anti-apoptotic, cell proliferation, oncogenic or pro-inflammatory in 10-day exposed murine HM. (**A**) Columns of graphs created by Graph Pad Prism software 7.0 depict mRNA phenotype in 10-day (two times per day) exposed HM to acidic bile (pH 3.0), pre-BAY 11-7082 + acidic bile (pH 3.0), and acidic bile (pH 3.0) + post-BAY 11-7082. mRNA phenotypes correspond to transcriptional expression ratios of NF-*κ*B-related genes *Bcl2*, *Rela, Egfr, Wnt5a*, *Stat3, Tnf, Il6* and *Ptgs2* comparing treated HM to saline treated HM, used as reference control, by real-time qPCR analysis (mRNA levels of each target gene were normalized to *Gapdh*). We observe an upregulation of mRNA expression of the analyzed genes in acidic bile-treated HM. Pre- or post-application of BAY 11-7082 reverses the acidic bile-induced mRNA phenotype. (**B**) Graphs, created by Graph Pad Prism 7.0 software, show transcriptional levels (normalized to *Gapdh*) for each analyzed gene, comparing HM exposed to acidic bile, application of BAY 11-7082 before or after acidic bile and saline-DMSO by real-time qPCR analysis (*p* value < 0.05; by *t* test; multiple comparisons by Holm-Sidak; data obtained from four analyzed samples). (**C**) Graph depicts the mRNA levels of all the analyzed genes, in acidic bile, pre- and post- treated by BAY 11-7082 HM. (^*^ *p* < 0.05; ^***^ *p* < 0.0005; by one-way ANOVA; Friedman test).

Quantitative PCR analysis was performed to reveal that the inhibitory effect of BAY 11-7082 reduced the NF-*κ*B related mRNA oncogenic profile when it was applied either before or after acidic bile. We observed that 10-day contact of HM to acidic bile induced a significant upregulation of *Rela, Stat3, Egfr, Bcl2*, *Wnt5a*, *Tnf, Il6* and *Ptgs2* relative to controls (saline-DMSO treated HM) (*p* < 0.05; multiple *t*-test) (Figure 3A and 3B) (Table 1), as in our previous findings [24]. However, the topical effect of BAY 11-7082 when it was applied either before or after acidic bile effectively inhibited its induced transcriptional activation of the analyzed genes. We found that HM pre- or post-treated by BAY 11-7082 produced significantly reduced mRNAs of *Rela, Stat3, Bcl2*, *Tnf, Wnt5a*, *Il6* and *Egfr* relative to HM exposed to acidic bile alone (Figure 3B) (Table 1 and Supplementary Table 1).

**Table 1:** Relative expression changes of acidic bile-related mRNA^‡^ oncogenic in topically pre- and post-treated murine HM with NF-κB inhibitor (BAY 11-7082)

	^‡^ACIDIC BILE vs. CNTL	^*^PRE-BAY vs ACIDIC BILE	^**^POST-BAY vs ACIDIC BILE
***Stat3***	186.2	–3336.0	–39.9
***Rela***	37.1	–55.5	–18.6
***Il6***	5.3	–2.8	1.2
***Ptgs2***	5.0	–1.9	1.0
***Wnt5a***	4.0	–57.4	–4.2
***Tnf***	2.0	–21.5	–6.7
***Bcl2***	1.6	–56.5	–4.6
***Egfr***	1.5	–1779.7	–16.7

^‡^Normalized mRNA levels, by qPCR; ^‡^Acidic bile: short-term application of bile at pH 3.0, twotimes per day for 10 days; ^*^Pre-BAY: pre-application of BAY 11-7082; ^**^Post-application of BAY 11-7082.

The administration of NF-*κ*B inhibitor before acidic bile resulted in a greater inhibitory effect on the transcriptional activation of analyzed genes than its administration after the acidic bile. This was identified by significantly lower mRNAs of *Rela, Stat3, Egfr, Bcl2*, *Wnt5a*, *Tnf, Il6* and *Ptgs2* in pre-treated relative to post-treated HM with BAY 11-7092 (Figure 3B) (Table 1). We also observed that overexpression of *Il6* and *Ptgs2* was only significantly affected by pre-application of NF-*κ*B inhibitor.

HM exposed to NF-*κ*B inhibitor before acidic bile produced a reversed mRNA phenotype relative to HM treated by acidic bile alone (Figure 3A and 3C). Treatment of HM with NF-*κ*B inhibitor after acidic bile exposure induced a similar effect, however, the post-treatment with BAY 11-7082 resulted in an inhibitory effect of fewer genes [*Stat3, Rela, Tnf, Wnt5a*, *Bcl2*, and *Egfr*] than its pre-application (Figure 3A and 3B) (Table 1; Supplementary Table 1).

Spearman analysis revealed significant correlations between NF-*κ*B inhibition-induced mRNAs of the oncogenic profile. Specifically, the Spearman test revealed a significant linear correlation between mRNA ratios of *Rela* and *Stat3*, as well as among *Egfr, Bcl2, Wnt5a,* and *Tnf* (*r* > 0.99, *p* < 0.05).

Overall, our analysis revealed that *in vivo* pre- or post-treatment of HM with NF-*κ*B inhibitor, significantly prevented the acidic bile-induced upregulation of a cancer-related profile [4, 7, 20, 26], as previously shown by the simultaneous topical co-administration of BAY 11-7082 with acidic bile on HM [24]. Administration of BAY 11-7082 before acidic bile resulted in a more intense inhibitory effect of acidic bile-induced molecular changes compared to its post-administration, as we had also recently shown *in vitro* [25].

### Inhibition of acidic bile-induced deregulation of oncogenic MicroRNA profile by *in vivo* topical exposure of HM to BAY 11-7082 either before or after acidic bile exposure

**Figure 4:**
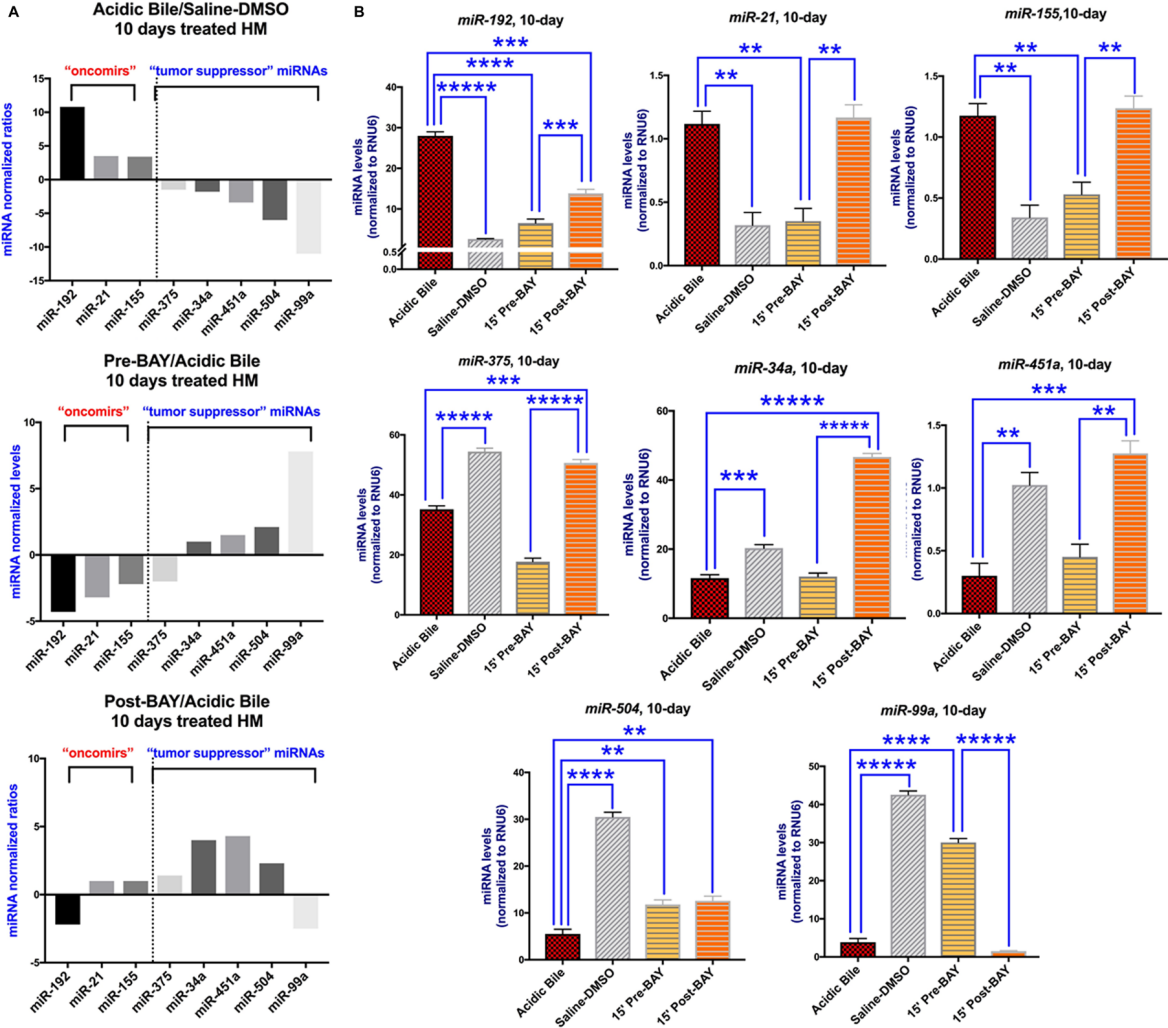
The *in vivo* pre- or post- topical application of BAY 11-7082 prevents the acidic bile-induced deregulation of cancer-related miRNA markers in 10-day exposed murine HM. (**A**) Columns of graphs created by Graph Pad Prism software 7.0 depict oncogenic miRNA phenotype (“oncomirs” and “tumor suppressor” miRNAs) in 10-day (two times per day) exposed HM to acidic bile (pH 3.0) vs. saline-DMSO treated HM, pre-BAY 11-7082 + acidic bile (pH 3.0) vs. acidic bile alone treated HM, and acidic bile (pH 3.0) + post-BAY 11-7082 vs. acidic bile alone treated HM (miRNA levels of each target miRNA marker were normalized to RNU6; by real-time qPCR analysis). We observe that pre- or post- application of BAY 11-7082 reverses the acidic bile-induced miRNA phenotype. (**B**) Graphs, created by Graph Pad Prism 7.0 software, reveal miRNA levels (normalized to *RNU6*) for each analyzed miRNA marker, comparing HM exposed to acidic bile alone, application of BAY 11-7082 before or after acidic bile and saline-DMSO, by real-time qPCR analysis (*p* value < 0.05; by *t* test; multiple comparisons by Holm-Sidak; data obtained from four analyzed samples).

BAY 11-7082 blocked the NF-*κ*B-related miRNA oncogenic phenotype when it was applied either before or after acidic bile. We observed that 10-day contact of HM to acidic bile compared to controls (saline-DMSO treated HM), induced a significant upregulation of “oncomirs” *miR-192, miR-21, miR-155,* and downregulation of “tumor suppressor” *miR-375*, *miR-34a, miR-451a*, *miR-504,* and *miR-99a,* as shown in our previous *in vivo* studies [7, 24], and linked to hypopharyngeal squamous cell carcinoma associated with biliary-reflux [20] (*p* < 0.05; multiple *t*-test) (Figure 4A and 4B) (Table 2). On the other hand, the topical effect of NF-*κ*B inhibitor when it was applied either before or after acidic bile effectively inhibited acidic bile-induced miRNA deregulations, producing a reversed miRNA phenotype, compared to HM exposed to acidic bile alone (Figure 4A) (Table 2 and Supplementary Table 2).

**Table 2:** Relative expression changes of acidic bile-related miRNA^‡^ oncogenic in topically pre- and post-treated murine HM with NF-κB inhibitor (BAY 11-7082)

	^‡^ACIDIC BILE vs. CNTL	^*^PRE-BAY vs ACIDIC BILE	^**^POST-BAY vs ACIDIC BILE
***“oncomirs”***			
***miR-192***	10.8	–4.3	–2.0
***miR-21***	3.5	–3.2	1.0
***miR-155***	3.4	–2.2	1.0
***“Tumor Suppressor”***			
***miR-375***	–1.6	–2.0	1.5
***miR-34a***	–1.8	1.0	4.0
***miR-451a***	–3.4	1.5	4.3
***miR-504***	–6.0	2.1	2.3
***miR-99a***	–11.1	7.0	–2.5

^‡^Normalized miRNA levels, by qPCR; ^‡^Acidic bile: short-term application of bile at pH 3.0, twotimes per day for 10 days; ^*^Pre-BAY: pre-application of BAY 11-7082; ^**^Post-application of BAY 11-7082.

Specifically, we found that HM pre- or post-treated by BAY 11-7082, produced significantly reduced levels of “oncomir” *miR-192*, as well as increased levels of “tumor suppressor” *miR-504*, relative to HM exposed to acidic bile alone (Figure 4B) (Table 2 and Supplementary Table 2).

We also observed that acidic bile-induced upregulation of “oncomirs” *miR-21* and *miR-155* was only affected by pre-application of NF-*κ*B inhibitor. This observation was confirmed by significantly lower levels of *miR-21 and miR-155*, in pre-treated HM with BAY 11-7082 relative to HM exposed to acidic bile alone or post-treated with BAY 11-7082 (Figure 4B) (Table 2). Also, the administration of NF-*κ*B inhibitor before acidic bile also resulted in a greater inhibitory effect on the “tumor suppressor” *miR-99a*, than its administration after acidic bile. This view was confirmed by significantly higher levels of *miR-99a* in pre-treated, relative to HM exposed to acidic bile alone or post-treated with BAY 11-7082 (Figure 4B) (Table 2 and Supplementary Table 2).

In addition, we also found that acidic bile-induced downregulation of “tumor suppressor” *miR-375* was only affected by post-application of NF-*κ*B inhibitor, again confirmed by significantly higher levels of *miR-375*, in post-treated HM relative to HM exposed to acidic bile alone or pre-treated with BAY 11-7082 (Figure 4B) (Table 2). The administration of NF-*κ*B inhibitor after acidic bile also resulted in a greater inhibitory effect on the “tumor suppressor” *miR-34a* and *miR-451a*, than its administration before acidic bile with significantly higher levels of *miR-34a and miR-451a,* in post-treated, relative to HM exposed to acidic bile alone or pre-treated with BAY 11-7082 (Figure 4B) (Table 2 and Supplementary Table 2).

Spearman analysis revealed significant correlations between NF-*κ*B inhibition-induced miRNA profiles, suggesting possible interactions among them. Specifically, the Spearman test revealed a significant positive correlation between *miR-21* and *miR-155, miR-451a* and *miR-34a* (*r* = 1, *p* < 0.05), and a reverse correlation between *miR-21* and *miR-99a,* and *miR-155* and *miR-99a* (*r* = –1, *p* < 0.05).

In summary, miRNA analysis revealed that both pre- or post-treatment of HM with a pharmacologic inhibitor of NF-*κ*B, significantly prevented and suppressed the acidic bile-induced upregulation and downregulation, respectively, of cancer-related miRNA phenotypes [5, 7, 20], as previously shown by the simultaneous topical co-administration of BAY 11-7082 with acidic bile on HM [24]. Administration of BAY 11-7082 before acidic bile resulted in a more intense inhibitory effect of acidic bile-induced miRNA deregulations compared to its post-administration (Figure 4A) (Table 2 and Supplementary Table 2), as we had also recently shown *in vitro* [25].

## DISCUSSION

Gastroesophageal reflux disease is characterized by reflux events that may occur randomly throughout the day while the duration and frequency of events may also vary within and across patients [27]. To synchronize a topically applied treatment regimen corresponding to each reflux event would not be clinically practical. In this inquiry we hypothesize that NF-*κ*B inhibitors administered either before or after bile exposure exert comparable effects of control. In recent publications we have documented the role of NF-*κ*B in acidic bile-induced malignant transformation of murine hypopharyngeal mucosa [4, 7]. The transcriptional effects of NF-*κ*B are shown to appear as early as 7 days, in the bile-related neoplastic process. We have also demonstrated that the acidic bile-induced activation of NF-*κ*B and its related oncogenic mRNA and miRNA phenotypes in murine hypopharyngeal mucosa, can be effectively prevented by the simultaneous topical co-application of pharmacologic NF-*κ*B inhibitor, BAY 11-7082 [24]. BAY 11-7082 is known to inhibit I*κ*B-α phosphorylation and its proteosomal degradation, blocking NF-*κ*B nuclear translocation and therefore preventing its binding to target genes that promote their transcriptional activation [28, 29]. Our previous *in vitro* explorations have strongly supported observations that BAY 11-7082 is an effective NF-*κ*B inhibitor of cancer-related profiles induced by acidic bile in hypopharyngeal primary cells not only when co-applied concurrently with acidic bile but also when administered either before or after acidic bile exposure [25].

Here, our novel findings document that *in vivo* topical application of BAY 11-7082 to HM, need not be precisely synchronized to bile exposure, in successfully blocking both the activation of NF-*κ*B and deregulation of cancer-related genes and specific miRNA markers [13–19, 30–42], previously linked *in vivo* to pre-malignant changes and invasive squamous cell carcinogenesis [4, 7]. Both short duration *in vivo* models of pre and post inhibitor application adequately reduce the oncogenic profiles caused by acidic bile, thus strongly supporting the effectiveness of models using intermittent as opposed to continuous therapy, and perhaps, of equal import, the efficacy of topical rather than systemic presentations of the NF-*κ*B inhibitor.

Our data further show that topical pre-administration of NF-*κ*B inhibitor on murine HM can be significantly more effective than its post-administration, in inhibiting the upregulation of NF-*κ*B transcriptional factor, *Rela,* anti-apoptotic or cell proliferation factors, *Bcl2, Stat3, Egfr, Wnt5a, Tnf, Il6* and *Ptgs2,* as well as “oncomirs” *miR-21, miR-155, miR-192* and “tumor suppressor” *miR-99a,* caused by acidic bile exposure. These observations suggest that deregulation of these cancer-related mRNA and miRNA phenotypes, previously linked to biliary-related hypopharyngeal neoplastic process and squamous cell carcinoma [7, 20], are directly promoted by acidic bile via NF-*κ*B shortly after its exposure and strongly suggest these molecules as potential biomarkers of early neoplastic events caused by bile reflux may be used as therapeutic targets.

Our current data are in line with our prior *in vivo* findings, providing the effectiveness of simultaneous co-administration of BAY 11-7082 [24], in suppressing *Stat3* and *Egfr* expression, both important contributors to HNSCC pathogenesis [13, 30], further emphasizing the need to develop a therapeutic strategy for targeting NF-*κ*B in head and neck malignancies. Cancer-related cytokines, such as *Tnf*, as well as *Wnt5a* [31, 32], an important mediator of an epithelial to mesenchymal transition (EMT) process [35] and previously shown to be rapidly induced by NF-*κ*B, are both successfully inhibited by either pre- or post- topical administration of BAY 11-7082. The observation that *Tnf* expression is more strongly reduced by pre-application of NF-*κ*B inhibitor compared to its post-application is in accordance with our recent *in vitro* data [25], supporting a direct inhibitory effect on acidic bile-induced *Tnf* transcriptional activation. Conversely, compared to our recent *in vitro* findings [25], the data presented here support the observation that acidic bile-induced activation of *Tnf* on HM cannot escape late NF-*κ*B inhibition by topical application of BAY 11-7082. The observation that either pre- or post- treatment by BAY 11-7082 significantly reduces the effect of acidic bile in upregulating *Bcl2* supports prior *in vitro* data [25] and the understanding that either early or late NF-*κ*B inhibition is capable of suppressing the acidic bile-induced anti-apoptotic pathways linked to tumorigenesis in exposed HM [7, 34]. In addition, the effectiveness of topical pre-application of BAY 11-7082 in inhibiting the acidic bile-induced transcriptional activation of cancer-related pro-inflammatory *Il6* [22, 23, 35] and *Ptgs2* [36], as similarly observed by its co-administration on HM [24], indicates their activation mediated by NF-*κ*B occurs early.

Administration of NF-*κ*B inhibitor, 15 minutes after the application of acidic bile shows minimal effects on acidic bile-induced transcriptional activation of *IL6* and *PTGS2*, as well as of *miR-21* and *miR-155*, and miR-99a, supporting the observation that bile induces a rapid pro-inflammatory effect in exposed HM, in line with preclinical and clinical observations [7, 19, 21]. However, it adequately blocks the acidic bile-induced overexpression of *Rela, Bcl2, Stat3, Egfr, Wnt5a,* and *Tnf,* as well as deregulation of *miR-192, miR-504*, *miR-451a, miR-375, and miR-34a*, previously associated with laryngopharyngeal cancer or biliary reflux-related hypopharyngeal carcinogenesis [7, 21, 39–42], in a manner similar to *in vivo* simultaneous co-administration of BAY 11-7082 [24]. Interestingly, both pre- or post-application of BAY 11-70782 significantly inhibits acidic bile-induced “upregulation of “oncomir” *miR-192*, previously linked with GERD [37], and downregulation of “tumor suppressor” *miR-504*, a promising target of hypopharyngeal squamous cell carcinoma [40], emphasizing their potential future use as possible therapeutic targets of bile-related mutagenic progression in the hypopharyngeal mucosa.

Immunohistochemical analysis shows significant reduction of nuclear positivity of p-NF-*κ*B in pre- and post-treated HM, mainly observed in cells of basal layer. This is in contrast to acidic bile treated HM, which produces an intense nuclear p-NF-*κ*B staining extending throughout its thickness, supporting a view that acidic bile induces a constitutive activation of NF-*κ*B which continues even after an initial acidic bile exposure. Our results further suggest that post-application of an NF-*κ*B inhibitor may be particularly effective on the constitutive component NF-*κ*B activation.

Microscopic examination of treated HM stained with cell proliferation marker Ki67 demonstrates that either pre or post-application of NF-*κ*B inhibitor, suppresses the cell proliferation or regeneration potential of mucosa promoted by acidic bile. The observation that pre or post NF-*κ*B inhibition reduces NF-*κ*B activation both in superficial layers of HM and in its regenerating basal layer implies that pre- or post- topical application of BAY 11-7082 is capable of globally preventing NF-*κ*B-induced cell proliferative events under the effect of acidic bile. This is in accordance with our previous observations [24], and supports the use of a suitable, topical NF-*κ*B inhibitor in effectively suppressing early preneoplastic changes [4, 5, 7].

Finally, based on our previous findings, long-term exposure of the hypopharyngeal mucosa to acidic bile progressively causes its malignant transformation, producing a progressive increase in the positivity of double-strand breaks (DSBs), oxidative DNA/RNA damage markers, overall p53 and nuclear NF-*κ*B protein levels, as well as the deregulation of NF-*κ*B-related oncogenic mRNA and miRNA phenotypes that are found to precede the histological evidence [7]. We do not yet know whether inhibition of acidic bile-induced NF-*κ*B, using BAY 11-7082, is capable of directly or indirectly influencing DNA stability in the form of oxidative DNA damage and DSBs, and/or p53 expression, according to previous studies [43–48]. Long-term exposure to BAY 11-7082 may further reveal how NF-*κ*B inhibition suppresses the initiation of cancer, which is the subject of active inquiry in our current research program.

## MATERIALS AND METHODS

All methods were carried out in accordance with guidelines and regulations of Yale University. The experimental protocol was approved by Yale Institutional Animal Care & Use Committee (IACUC) (https://your.yale.edu/research-support/animal-research) (approved protocol 11039 by IACUC of Yale University).

### *In vivo* model

As established in our previous *in vivo* model [4, 5, 7, 24], we used C57BL/6J (Mus, Musculus; Jax mice, Jackson Laboratory USA). A full description is presented in supplementary methods section (Supplementary Material). Briefly, 20 males and 20 females were randomly divided in the following groups [8 mice (4 males + 4 females) per group]: (i) acidic bile-treated group (pH 3.0), at concentrations previously described [50, 51] and described in supplementary methods (Supplementary Material), (ii) pre-treated group (treatment of HM with 0.25 μmol of BAY 11-7082 (~0.75 μmol per day) [24] 15 min before acidic bile exposure [25]), and (iii) post-treated group (treatment of HM with 0.25 μmol of BAY 11-7082 (~0.75 μmol per day) 15 min after acidic bile exposure [26]), (iv) saline-DMSO (pH 7.0) treated HM, and (v) an untreated control group (negative control). The HM of treated groups was exposed two times per day (with an interval of 6 hours during which animals had access to drinking water, ensuring adequate wash out between treatments) for 10 days (20 applications), using a plastic feeding tube [4, 5, 7, 24].

### Tissue examination

Three- to 4-μm-thick tissue sections of formalin-fixed and paraffin-embedded HM from experimental and control groups were stained with hematoxylin and eosin and examined by light microscopy, to exclude histological signs of toxicity (hemorrhagic lesions or ulceration) caused by topical supplication procedure.

### Immunohistochemical (IHC) analysis

IHC analysis for p-NF-*κ*B (p65 S536) and Ki67 (cell proliferation marker) was performed on hypopharyngeal tissue sections from all experimental and control specimens to detect nuclear proteins in hypopharyngeal mucosal layers, as previously described [4, 5, 7, 20, 24], and summarized in the Supplementary Material.

Nuclear p-NF-*κ*B (p65 S536) and Ki67 protein levels in experimental and controls were expressed as positive nuclei to total number (defined as positivity) derived from two independent images per tissue section (at least four tissue sections per group) (mean ± SD by multiple *t*-test).

### Gene expression and miRNA analysis

We isolated total RNA from murine HM (four animals per group) exposed to acidic bile at pH 3.0, pre- or post-treated with NF-*κ*B inhibitor, and corresponding controls, to evaluate by reverse transcription and quantitative real-time polymerase chain reaction (qPCR) analysis the mRNA levels of target genes, *Rela, Bcl2*, *Tnf, Egfr, Stat3, Wnt5a*, *Il6* and *Ptgs2,* previously identified in our prior studies and described in Supplementary Table 3 [4, 7, 20, 24, 25], relative to the reference gene (*Gapdh*) (ΔΔCt) as further described in Supplementary Material.

To analyze *miR-21, miR-155, miR-192, miR-34a, miR375, miR-451a, miR-99a,* and *miR-504* levels, we performed reverse transcription synthesis of miRNAs from total RNA using miScript II RT kit (Qiagen, Louisville, KY), following by qPCR analysis, using specific primers for mouse genome (miScript Primer Assays; Qiagen^®^, KY, USA) Supplementary Table 4, as described previously [5, 7, 20, 23–25]. We used CFX96^TM^ (Bio-Rad) software to estimate relative expression levels for each specific target gene or miRNA relative to the reference controls (*RNU6*) (ΔΔCt).

### Statistical analysis

GraphPad Prism 6 software and one-way analysis of variance (ANOVA) (Friedman test and Dunn’s multiple-analysis test were used to determine significance defined as *p* values <0.05). A *t*-test analysis (multiple comparisons by Holm-Sidak) was used to reveal statistically significant changes in protein, mRNA and miRNA expression levels among the studied groups. Spearman non-parametric test was used to estimate the correlation coefficient between gene expression and miRNA levels in the studied groups (*p* values <0.05).

## CONCLUSIONS

Our novel findings demonstrate that topical pre- or post-administration of pharmacologic inhibitor NF-*κ*B 15 min before, or 15 min after acidic bile exposure successfully prevents and suppresses oncogenic molecular events produced in murine HM. In general, our current observations strongly support the view that topical NF-*κ*B inhibition, without precise synchronization to acidic bile exposure, may be clinically feasible in preventing acidic bile-induced oncogenic molecular changes. Although, there may be limitations of BAY 11-7082 for clinical application due to its inherently global toxicities, other forms of topical inhibitor are worthy of future investigation. Dietary NF-*κ*B inhibitors, such as curcumin, for example, provide a wide range of opportunities for therapeutic clinical intervention. Doukas et al., recently demonstrated that topically applied curcumin, even if it is not precisely synchronized with the application of acidic bile, can effectively suppress the induced mRNA oncogenic phenotype in murine hypopharyngeal mucosa [49]. Equally important, our novel findings reveal several gene products and miRNA molecules as possible biomarkers of early oncogenic changes caused by acidic bile reflux, including *Bcl2, Stat3, Egfr, Wnt5a, Tnf, Il6* and *Ptgs2, miR-21, miR-155, miR-192*, *miR-99a, miR-375, miR-34a, miR-451a,* and *miR-504,* as well as strongly suggesting the use of *Stat3, Egfr, Wnt5A, Tnf, Bcl2, miR-192 and miR-504,* as promising therapeutic targets in future preclinical and clinical trials in combination with NF-*κ*B inhibition.

## SUPPLEMENTARY MATERIALS


