# Supplementary material for: The temporal effects of topical NF-κB inhibition, in the in vivo prevention of bile-related oncogenic mRNA and miRNA phenotypes in murine hypopharyngeal mucosa: a preclinical model
Source: Oncotarget. 2020 Sep 1;11(35):3303–14. doi: 10.18632/oncotarget.27706 (PMC7476734; doi:10.18632/oncotarget.27706)
Supplement: Supplementary file 1 [file oncotarget-11-3303-s001.pdf]

The temporal effects of topical NF-*κB* inhibition, in the *in vivo* prevention of bile-related oncogenic mRNA and miRNA phenotypes in murine hypopharyngeal mucosa: a preclinical model

SUPPLEMENTARY MATERIALS

In vivo model

We used Mus Musculus, mouse strain C57BL/6J (Jax mice, Jackson Laboratory USA) [20 males and 20 females; 8 mice (4 males + 4 females) per group]. Performing repetitive procedures, in parallel, we topically exposed murine HM to (i) a mixture of bile salts (10 mmol/l in buffered saline) (~5 μmol per day) at molar concentrations previously described and considered to be close to "physiologic" [43, 44], at pH 3.0, (ii) pre-application and (iii) post-application of 0.25 μmol of BAY 11-7082 (~0.75 μmol per day) (Calbiochem; EDM Millipore Corp.) [24]. This was done two times per day (with an interval of 6 hours in which the animals had access to drinking water, ensuring adequate wash out between treatments) for 10 days (20 applications), using a plastic feeding tube [4, 7, 24]. Acidic pH of 3.0 was selected, as previously described by Vageli D, et al., to induce a marked NF-*κ*B activation and overexpression of a cancer mRNA phenotype in murine hypopharyngeal mucosa [4, 7].

The experimental procedures included:

(i) Topical application of a mixture of conjugated bile salts (400 μM) (Glycocholic acid:taurocholic acid:glycochenodeoxycholic acid:taurodeoxycholic acid: glycodeoxycholic acid: taurodeoxycholic acid at molar concentration 2-:3:15:3:6:1) (Sigma, St. Louis, MO; Calbiochem, San Diego, CA, USA) in buffered saline brought to pH 3.0 with 1M HCl (using a pH meter) to HM, as previously described [3, 4].

(ii) Topical application of 0.25 μmol of BAY 11-7082 Calbiochem; EDM Millipore Corp.) in buffered saline at pH 7.0, 15 minutes preceding the topical application of acidic bile at pH 3.0 [25].

(iii) Topical application of 0.25 μmol of BAY 11-7082 in buffered saline at pH 7.0 15 min after the topical application of acidic bile at pH 3.0.

The control groups included repetitive topical applications with (i) saline at a pH 7.0 (reference control for the mechanical effect of the feeding tube on HM) combined with the NF-*κ*B inhibitor vehicle (DMSO), and (ii) an untreated control group (negative control).

At the end of the procedures, experimental and control animals were euthanized using CO_2 (IACUC euthanasia policy and guidelines). The euthanized animals were kept on ice for dissection of HM tissue fragments. The HM from four animals (two males and two females) of each group, were placed immediately into 10% neutral buffered formalin (Thermo Fisher Scientific, Middletown, VA) to be submitted for embedding in paraffin blocks (Yale Pathology Facilities). The remaining tissue fragments from each experimental and control groups were immersed in RNA stabilization solution (RNAlater, Life Technologies, Grand Island, NY) and stored at –80°C for RNA isolation.

Immunohistochemical (IHC) analysis for NF-*κ*B (p65 Phosphorylated at Ser536)

We performed IHC analysis for p-NF-*κ*B (p65 S536) to explore the effect of topical pre- or post- application of NF-*κ*B inhibitor to respectively prevent or suppress, the acidic bile-induced NF-*κ*B activation [increased nuclear positivity of p-NF-*κ*B (p65 S536)], in 10-day treated murine HM [24]. We performed chromogenic IHC using immunoperoxidase (DAB peroxidase substrate) for p-NF-*κ*B (1:80 of anti-NF-*κ*B rabbit polyclonal anti-phospho-p65 Ser536, AbD Serotec, BIORAD, CA, USA) on hypopharyngeal tissue sections from all experimental and control specimens as previously described [4, 7, 24], to detect nuclear proteins in the basal/parabasal/suprabasal cells of HM. We analyzed the slides using a Leica light microscope, and captured images using Aperio CS2. The images were analyzed by Image Scope software (Leica Microsystems, Buffalo Grove, IL) that generated algorithms illustrating the mucosal and cellular compartments of p-NF-*κ*B staining.

Nuclear p-p65 (S536) protein levels in acidic bile treated HM, HM pre- or post- treated with NF-*κ*B inhibitor, saline-DMSO treated HM, and untreated controls, were expressed as ratios of positive nuclei to

total number (defined as positivity) derived from two independent images per tissue section (at least four tissue sections per group; mean ± SD by multiple *t* test).

Immunohistochemical (IHC) analysis for Ki67

We performed IHC analysis for Ki67, a cell proliferation marker, in hypopharyngeal tissue sections from all experimental and control specimens. This was done to explore the effect of 10-day topical exposure of HM to acidic bile (pH 3.0), on increasing regenerative activity of mucosal basal/parabasal layers, and consequently the impact of pre- and post- application of NF-κB inhibitor on reducing that effect. We used chromogenic IHC with immunoperoxidase (DAB peroxidase substrate) and 1:200 dilutions of anti-Ki67 (rabbit mAb, SP6, Thermo Scientific Lab Vision, UK) to detect nuclear protein in basal/parabasal cells of HM. We analyzed the slides using a Leica light microscope and captured images using Aperio CS2. The images were analyzed by Image Scope software (Leica Microsystems, Buffalo Grove, IL) that generated algorithms illustrating the mucosal and cellular compartments by Ki67 staining.

Nuclear Ki67 protein levels in acidic bile treated HM, and HM pre- or post- treated with NF-κB inhibitor, as well as saline-DMSO treated HM, and untreated controls, were expressed as positive nuclei to total number (defined as positivity) derived from two independent images per tissue section (at least four tissue sections per group) (mean ± SD by multiple *t* test).

Quantitative real-time polymerase chain reaction

Gene expression analysis

Total RNA was isolated from tissue specimens using RNeasy mini kit (Qiagen®, KY, USA), in order to perform real time qPCR. RNA quality was determined by absorption ratios at 260/280 nm (> 2.0) and concentration ratios by absorption at 260 nm using a NanoDrop™ 1000 spectrophotometer (Thermo Scientific). Reverse transcription to cDNA was performed using Whole Transcriptome kit (Qiagen®, KY, USA), following the manufacturer's instructions. The real time qPCR analysis (Bio-Rad real-time thermal cycler CFX96™) was performed using specific primers for mouse genome as indicated in Supplementary Table 3 and iQ™ SYBR® Green Supermix (BIO-RAD, CA, USA). Target genes included *Rela, Bcl2, Egfr, Tnf, Wnt5a, Stat3, Il6 and Ptgs2*, while *Gapdh* was used as a reference control gene (QuantiTect® primers assay, Qiagen®, KY, USA), to detect transcriptional expression levels of acidic bile-induced NF-κB related genes previously identified in our prior studies [4, 5, 7, 20, 24]. PCR assays were performed in 96-well plates and each sample was assayed in triplicate. Q-real time PCR data was analyzed by CFX96™ software (Bio Rad, CA, USA).

miRNA analysis

We performed miRNA analysis to determine the expression levels of "oncomirs" and 'tumor suppressor" miRNA specific markers in 10-day exposed-HM to acidic bile alone, pre or post treated with BAY 11-7082, and saline-DMSO treated controls. Specifically, we analyzed the expression of "oncomirs" *miR-21, miR-155, and miR-192*, and "tumor suppressors" *miR-34a, miR-375, miR-451a, miR-504*, and *miR-99a*, using primers for target-miRNAs of mouse genome (miScript Primer Assays, Qiagen®, KY, USA) and normalization control small RNA [snRNA *RNU6B* (*RNU6-2*)], as previously described [5, 7, 20, 23–25] (Supplementary Table 4). Briefly, miScript II RT kit (Qiagen, Louisville, KY) was used to perform reverse transcription synthesis of miRNAs from total RNA (isolated for qPCR analysis as described above) according to the manufacturer's instructions and we estimated relative expression levels (target miRNA/RNU6B) for each specific miRNA marker, in each experimental and control group (CFX96™ software; Bio-Rad, CA, USA).

Supplementary Table 1: Transcriptional levels of NF-κB related genes with oncogenic function in murine hypopharyngeal mucosa (HM)

Target gene/ *Gapdh** ($\Delta\Delta^{CT}$)	Acidic Bile	**DMSO	$^{\#}$Pre-BAY	$^{¥}$Post-BAY
Bcl2	2.70E-02	1.75E-02	4.78E-04	5.85E-03
Rela	3.11E-02	8.40E-04	5.61E-04	1.68E-03
Egfr	3.60E-04	2.44E-04	2.02E-07	2.15E-05
Stat3	2.70E-04	1.45E-06	8.09E-08	6.77E-06
Il6	2.80E-04	5.31E-05	1.00E-04	3.26E-04
Wnt5a	8.70E-04	2.24E-04	1.51E-05	2.08E-04
Tnf	6.25E-03	3.05E-03	2.90E-04	9.37E-04
Ptgs2	6.33E-02	1.32E-02	3.27E-02	6.29E-02

*normalization of mRNA levels using *Gapdh*; **Saline-DMSO treated HM; $^{\#}$Pre-BAY 11-7-082+Acidic bile treated HM; $^{¥}$Acidic Bile+Post-BAY 11-7082 treated HM.

Supplementary Table 2: Normalized expression levels of "oncomirs" and "tumor suppressor" miRNAs in murine hypopharyngeal mucosa (HM)

Target miRNA/ *RNU6** ($\Delta\Delta^{CT}$)	Acidic Bile	**DMSO	$^{\#}$Pre-BAY	$^{¥}$Post-BAY
miR-192	2.80E+01	2.59E+00	6.52E+00	1.39E+01
miR-21	1.12E+00	3.19E-01	3.51E-01	1.17E+00
miR-155	1.17E+00	3.41E-01	5.30E-01	1.24E+00
miR-375	3.52E+01	5.44E+01	1.77E+01	5.26E+01
miR-34a	1.16E+01	2.03E+01	1.21E+01	4.67E+01
miR-451a	3.00E-01	1.02E+00	4.51E-01	1.28E+00
miR-504	5.52E+00	3.30E+01	1.18E+01	1.26E+01
miR-99a	3.84E+00	4.25E+01	3.00E+01	1.54E+00

*normalization of miRNA levels using *RNU6*; **Saline-DMSO treated HM; $^{\#}$Pre-BAY 11-7-082+Acidic bile treated HM; $^{¥}$Acidic Bile+Post-BAY 11-7082 treated HM.

Supplementary Table 3: Mouse genes (targets and *GAPDH*) and their detected transcripts, analyzed by real time qPCR, in murine HM

Gene (mouse)	Detected transcripts	Amplicon length (bp)
Gapdh	NM_008084	144
	NM_001289726	
Rela	NM_009045	82
Stat3	NM_011486	99
	NM_213659	
	NM_213660	
Wnt5a	NM_001256224	130
	NM_009524	
Bcl2	NM_009741	80
Tnf	NM_013693	112
	NM_001278601	
Egfr	NM_007912	68
	NM_207655	
Il6	NM_031168	128
Ptgs2	NM_011198	95

Supplementary Table 4: Mouse mature miRNAs (targets) and reference *RNU6-2* small RNA control, analyzed by real time qPCR, in murine HM

miRNA (mouse)	Target mature miRNA, Sanger Accession)
miR-21a	mmu-miR-21a-5p, MI0000569
miR-155	mmu-miR-155-5p, MI0000177
miR-192	mmu-miR-192-5p, MI0000551
miR-375	mmu-miR-375-3p, MI0000792
miR-34a	mmu-miR-34a-5p, MI0000584
miR-451a	mmu-miR-451a, MI0001730
miR-504	mmu-miR-504-5p, MI0005515
miR-99a	mmu-miR99a-5p, MI0000146
**Small RNA**	**Control**
RNU6	U6 small nuclear RNA, ENSMUSG00000095132
